# Y-chromosomal connection between Hungarians and geographically distant populations of the Ural Mountain region and West Siberia

**DOI:** 10.1038/s41598-019-44272-6

**Published:** 2019-05-24

**Authors:** Helen Post, Endre Németh, László Klima, Rodrigo Flores, Tibor Fehér, Attila Türk, Gábor Székely, Hovhannes Sahakyan, Mayukh Mondal, Francesco Montinaro, Monika Karmin, Lauri Saag, Bayazit Yunusbayev, Elza K. Khusnutdinova, Ene Metspalu, Richard Villems, Kristiina Tambets, Siiri Rootsi

**Affiliations:** 1 Institute of Genomics, Estonian Biocentre, University of Tartu, Tartu, 51010 Estonia; 2 Hungarian Natural History Museum, Department of Anthropology, Budapest, 1083 Hungary; 3 Eötvös Loránd University Budapest, Department of Finno-Ugric Studies, Budapest, 1088 Hungary; 4 Pázmány Péter Catholic University, Faculty of Humanities and Social Sciences, Department of Early Hungarian and Migration Period Archaeology, Piliscsaba, 2087 Hungary; 5 Laboratory of Ethnogenomics, Institute of Molecular Biology of National Academy of Sciences, Yerevan, 0014 Armenia; 6 Institute of Biochemistry and Genetics, Ufa Scientific Center of RAS, Ufa, 450054 Russia; 7 Department of Genetics and Fundamental Medicine, Bashkir State University, Ufa, 450054 Russia; 8 Department of Evolutionary Biology, Institute of Molecular and Cellular Biology, University of Tartu, Tartu, 51010 Estonia

**Keywords:** Evolution, Population genetics

## Abstract

Hungarians who live in Central Europe today are one of the westernmost Uralic speakers. Despite of the proposed Volga-Ural/West Siberian roots of the Hungarian language, the present-day Hungarian gene pool is highly similar to that of the surrounding Indo-European speaking populations. However, a limited portion of specific Y-chromosomal lineages from haplogroup N, sometimes associated with the spread of Uralic languages, link modern Hungarians with populations living close to the Ural Mountain range on the border of Europe and Asia. Here we investigate the paternal genetic connection between these spatially separated populations. We reconstruct the phylogeny of N3a4-Z1936 clade by using 33 high-coverage Y-chromosomal sequences and estimate the coalescent times of its sub-clades. We genotype close to 5000 samples from 46 Eurasian populations to show the presence of N3a4-B539 lineages among Hungarians and in the populations from Ural Mountain region, including Ob-Ugric-speakers from West Siberia who are geographically distant but linguistically closest to Hungarians. This sub-clade splits from its sister-branch N3a4-B535, frequent today among Northeast European Uralic speakers, 4000–5000 ya, which is in the time-frame of the proposed divergence of Ugric languages.

## Introduction

**Figure 1:**
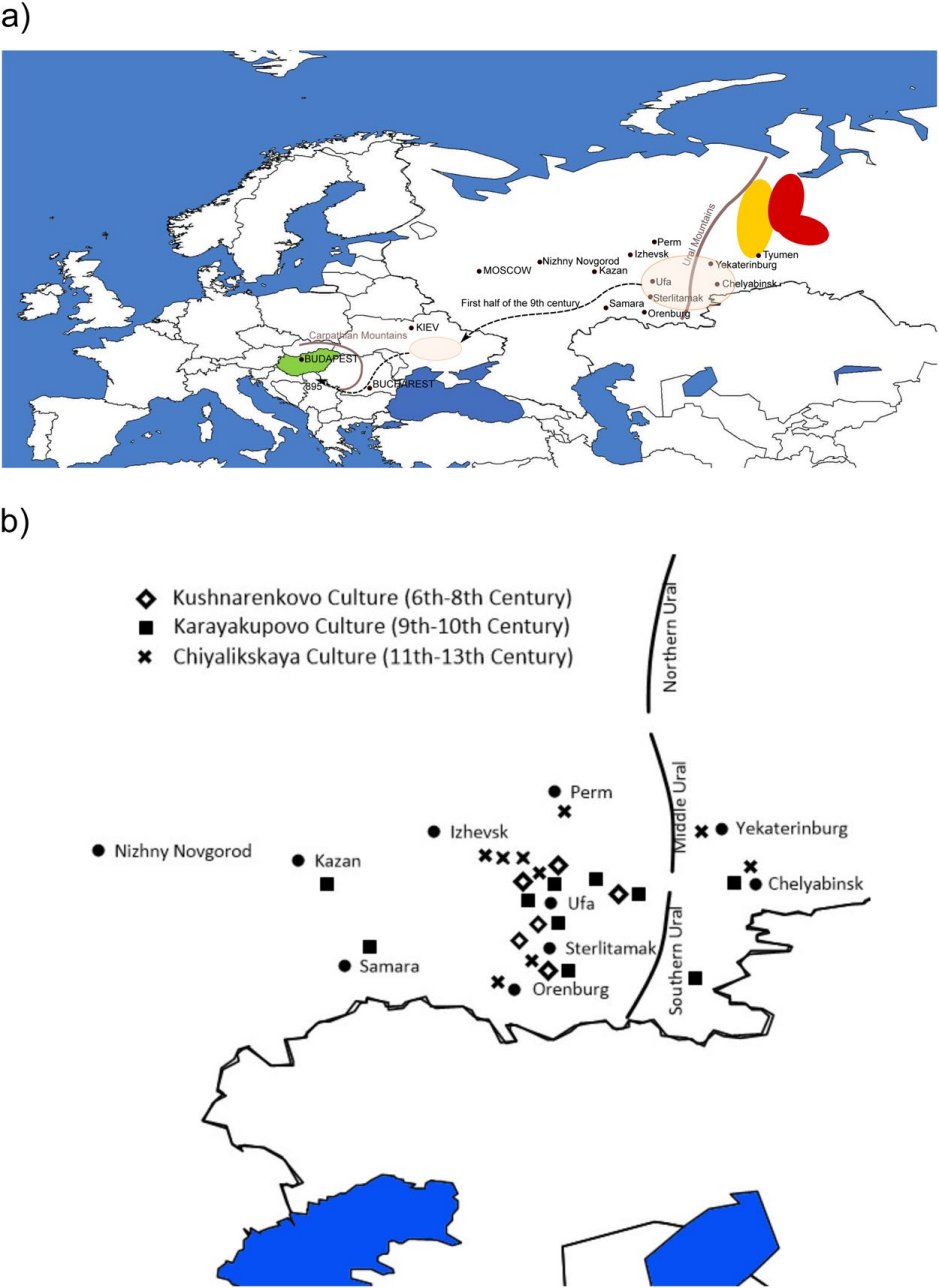
(**a**) Map of Western Eurasia and the putative migration route of early Hungarians based on archaeological data. Hungary is shown in green. Geographic locations of Mansis and Khantys are indicated with yellow and red, respectively. The pink transparent colour marks the geographic regions where archaeological evidence of Hungarian ancestors has been found. (**b**) Volga-Ural archaeological cultures from 6th to 13th century. Background maps from Surfer® (v.8, Golden Software, Inc, Golden, CO, USA).

The Uralic languages cover today a wide territory of North Eurasia from West Siberia in the east to Northeast Europe in the west. Hungarians with about 13 million speakers^1^ are the largest Uralic speaking group in the world^2^, who today reside in Central Europe (Fig. 1a) far apart from the rest of the members of their language family. Linguistically closest to Hungarians are geographically very distant West Siberian Mansi and Khanty (Fig. 1a), with whom they belong to the Ugric branch of the Uralic linguistic family^2–4^. In addition, the Hungarian language has been intensively influenced by several Turkic languages during the second half of the first millennium AD^5^. Agreeing with the linguistic results most of the archaeologists propose that the putative homeland of the ancestors of the Hungarian speaking population must have been in West Siberia^6–9^ (Fig. 1a).

Despite of the eastern roots of the Hungarian language the present-day gene pool of Hungarians is very similar to the neighbouring non-Uralic speaking Central Europeans according to autosomal^10,11^, Y-chromosomal (chrY)^12–16^ and mitochondrial DNA (mtDNA) data^13,17^. One of the main results of the study by Tambets *et al*.^10^ was that the Hungarians differ from the majority of Uralic-speakers – they do not show any specific link with their linguistic relatives compared to their non-Uralic neighbours in Central Europe. These results are in line with the earlier observations of anthropologists who have suggested that since the forefathers of Hungarians arrived in the Carpathian Basin at the end of the 9^th^ century^6,18^, the population has changed significantly during the demographic processes that homogenized different ethnic groups in the area within the last 1100 years^19^. Anthropological differences detected among ancient Hungarians from different macro-regions in the Carpathian Basin were interpreted as a possible reflection of their heterogeneous geographical origin and relatively recent admixture of ancient Hungarian groups^20^. Archaeogenetic studies also confirm the admixed genetic background of the early Hungarians. Comparing the Hungarian Conqueror mtDNA dataset to a large modern-day population dataset and archaeogenetic database, researchers found strong genetic affinities towards modern populations of Inner Asia, North and East Europe, Central Russia, and Late Bronze Age populations of the Baraba region, situated between the rivers Ob and Irtis^21^. Also, most researchers agree that the size of the Avar population, who resided in the Carpathian Basin in the 8–9th century AD, was unequivocally greater than the number of putative Hungarian ancestors^22^. It is notable, that the mtDNA gene pool of Avar “commoners” and mixed Avar-Slavic cemeteries showed significantly lower genetic distances toward medieval European populations than Asian populations, indicating that the genetic imprint of the Inner Asian Avar elite through their mtDNA was rather weak in those populations^23,24^. In addition, an ancient DNA study dealing with 6^th^ century barbarian migrations shows that Y-chromosomes of 21 ancient individuals from a Szólád cemetery (Hungary) belong to predominantly Central and Southern European haplogroups (E, I1, I2a2, T, R1a and R1b)^25^. Considering the possibility of sometimes intensive migration to Carpathian Basin from the neighbouring regions^26^, it is reasonable that present-day gene pool of Hungarians has become very similar to neighbouring populations. However, it is an open issue if there is any trace in the recent Hungarian gene pool reflecting their possible homeland in the East.

Certain chr-Y lineages from haplogroup (hg) N have been proposed to be associated with the spread of Uralic languages^27^. So far, hg N3 has not been reported for Indo-European speaking populations in Central Europe^14,28–30^, but it is present among Hungarians, although the proportion of hg N in the paternal gene pool of present-day Hungarians is only marginal (up to 4%) compared to other Uralic speaking populations^27^. It has been shown earlier that one of the sub-clades of hg N – N3a4-Z1936 – could be a potential link between two Ugric speaking populations: the Hungarians and the Mansi^31^. It is also notable that some ancient Hungarian samples from the 9^th^ and 10^th^ century Carpathian Basin belonged to this hg N sub-clade^32^: Three Z1936 samples were found in the Upper-Tisza area (Karos II, Bodrogszerdahely/Streda nad Bodrogom) and two in the Middle-Tisza basin cemeteries (Nagykörű and Tiszakécske). The haplotype of the Nagykörű sample is identical with one contemporary Hungarian sample from Transylvania that tested positive for B545 marker downstream of N3a4-Z1936^32^. Similar findings come from the maternal gene pool of historical Hungarians: the analyses of early medieval aDNA samples from Karos-Eperjesszög cemeteries revealed the presence of mtDNA hgs of East Asian provenance^21^.

Archaeogenetic studies based on mtDNA haplotypes have shown that ancient Hungarians were relatively close to contemporary Bashkirs^33^ who are a Turkic speaking population residing in the Volga-Ural region. Another study reported excessive identical-by-descent (IBD) genomic segments shared between the Ob-Ugric speaking Khantys and Bashkirs but a moderate IBD sharing between Turkic speaking Tatars and their neighbours including Bashkirs^34^. According to this study the gene pool of Bashkirs is a mixture of Turkic, Ugric and Indo-European contributions. The complexity of genetic composition of Bashkirs was shown by Yunusbaev *et al*.^35^, both in autosomal and in chrY data.

In this study we use, for the first time, the chrY high coverage sequencing data of Hungarian samples together with samples from the populations from regions of the Ural Mountains and West Siberia. We refine the phylogeny of hg N, estimate the divergence times of sub-clades of hg N3a4 and, together with a large genotyping dataset, reveal the spatio-temporal distribution pattern of hg N3a4. This lineage is the genetic link between present-day Hungarians and populations from the Ural region and West Siberia, the proposed region of origin for the Hungarian language.

## Results

### Phylogenetic tree of hg N3a4 and coalescence ages of major sub-clades

**Figure 2:**
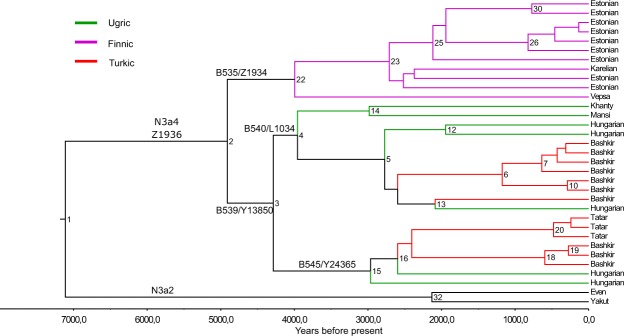
Phylogenetic tree of hg N3a4. Phylogenetic tree of 33 high coverage Y-chromosomes from haplogroup N3a4 was reconstructed with BEAST v.1.7.5 software package. We used 8 sequences published in Karmin *et al*.^63^, 6 sequences published in Ilumäe *et al*.^27^, 2 sequences published in Wong *et al*.^64^ and 17 new sequences from this study. Two N3a2 samples were used as an outgroup to estimate coalescent times. Internal node numbers on the branches (not including nodes with low posterior values), sub-clade names and population names on the tips are indicated. Branches are coloured according to language affiliations. Number of branch-defining mutations and marker names are presented in Supplementary Fig. S4. All SNPs characterizing the clades (nodes) are presented in Supplementary Table S1. Age estimates can be found in Supplementary Table S2.

To reconstruct the phylogeny of hg N3a4-Z1936 and refine its inner structure we used 33 high-coverage chrY sequences, including those of 5 Hungarians (Fig. 2). All variants accumulated on the branches are listed in Supplementary Table S1, the age estimates of N3a4 sub-clades are shown on Fig. 2 and presented in Supplementary Table S2.

Phylogenetic tree of hg N3a4 has two main sub-clades defined by markers B535 and B539 that diverged around 4.9 kya (95% confidence interval [CI] = 3.7–6.3 kya). Inner sub-clades of N3a4-B539 (defined by markers B540 and B545) split 4.2 kya (95% CI = 3.0–5.6 kya). Further sub-clades of B540, one containing Y-chromosomes of a Khanty and a Mansi and other containing chrY’s of Hungarians and Bashkirs (Fig. 2, Supplementary Table S2) split around 3.9 kya (95% CI = 2.8–5.2 kya). The expansions of B540 and B545 started around the same time about 2.7–2.9 kya (B540-PH573/L1442: 95% CI = 1.8–3.7 kya; B540-Y28538: 95% CI = 2.0–4.1 kya; B545: 95% CI = 2.0–4.1 kya) (Supplementary Table S2). The phylogenetic tree reveals that all five Hungarian samples belong to N3a4-B539 sub-clade that they share with Ob-Ugric speaking Khanty and Mansi, and Turkic speaking Bashkirs and Tatars from the Volga-Ural region. Hungarian and Bashkir chrY lineages belong to both sub-clades of N3a4-B539 (Fig. 2).

### Geographic distribution of genotyped hg N sub-lineages

Although, the frequency of hg N among modern Hungarians is only marginal, this small, but very intriguing from historical point of view, portion of paternal lineages belongs to N3a4 clade.

**Figure 3:**
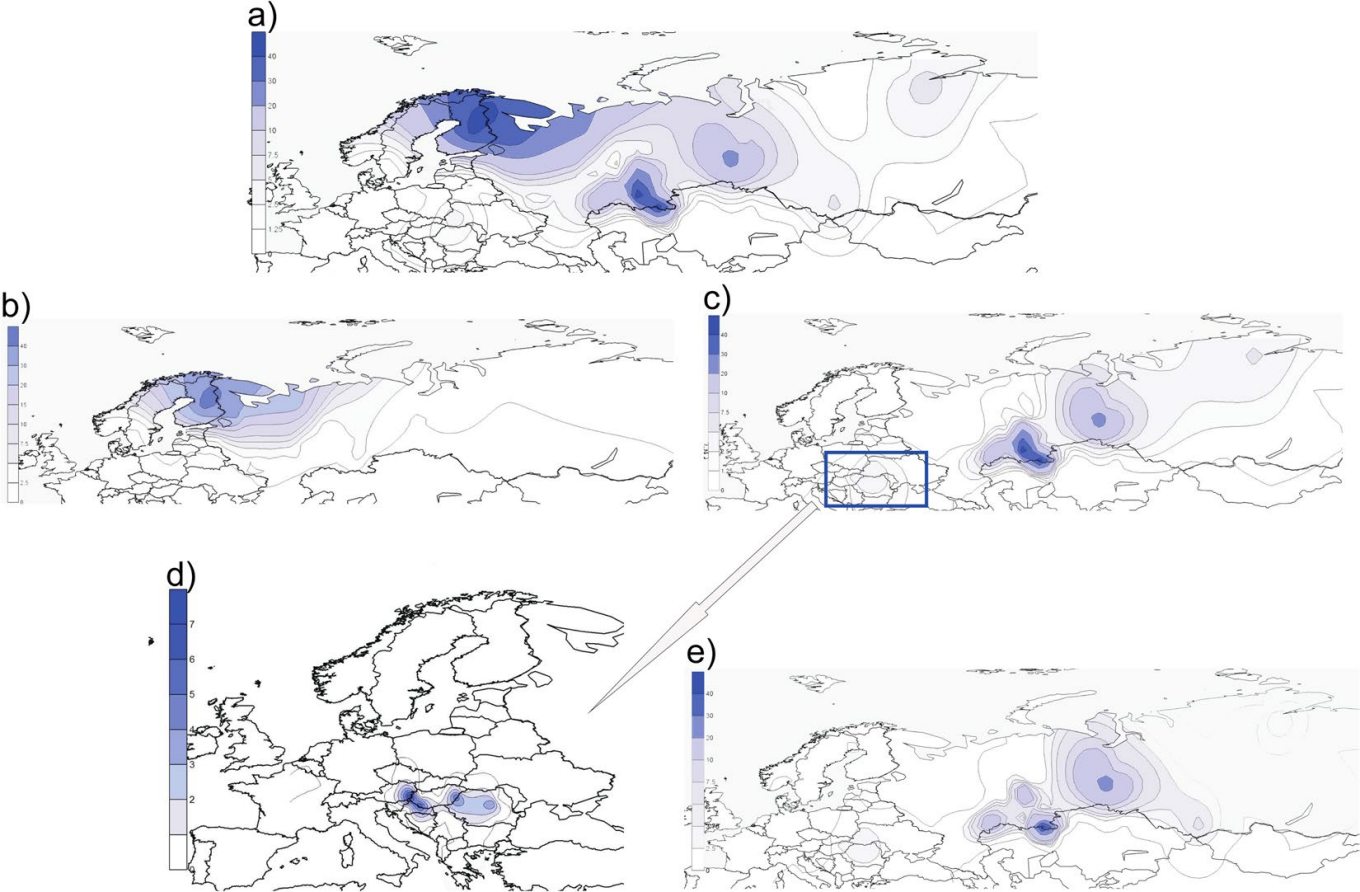
Frequency(%) distribution maps of individual sub-clades of hg N3a4. (**a**) Map of the entire hg N3a4. (**b**,**c**) Maps of N3a4 sub-clades defined by B535 and B539, respectively. (**d**) The local snapshot of B539 is a subsample of points presented on panel (c) showing the N3a4-B539 distribution among Hungarian speakers. Note that the scales of the maps are different. (**e**) Map of N3a4-B540/L1034. All the maps were created with Surfer v.8. Data points used for generating the maps are given in Supplementary Tables S3 and S4 for local B539 map. Background maps from Surfer® (v.8, Golden Software, Inc, Golden, CO, USA).

To test the presence and proportions of hg N3a4 lineages in a more comprehensive sample set and with a higher phylogenetic resolution level compared to earlier studies^14–16,31^, we analysed the genotyping data of about 5000 Eurasian individuals, including West Siberian Mansi and Khanty who are linguistically closest to Hungarians (Supplementary Fig. S1). Genotyping confirms that the N3a4-B539 clade found among Hungarians, Bashkirs and Tatars is also shared with Ugric speaking Mansis and Khantys (Supplementary Table S3), matching the findings of Feher *et al*.^31^ and showing that B539 clade is common for all Ugric speaking populations. To visualize the geographic spread of hg N3a4 and its sub-clades we used genotype frequencies **(**Supplementary Tables S3 and S4) to construct distribution maps (Fig. 3).

N3a4 has two frequency peaks (Fig. 3a) – one in Northeast Europe where mostly N3a4-B535 is found (Fig. 3b), and the other in southern slopes of the Ural Mountains where N3a4-B539 is prevalent (Fig. 3c). There is a clear difference in geographic distribution patterns of these two hg N3a4 sub-clades. Hg N3a4-B535 (Fig. 3b) is common mostly among Finnic (Finns, Karelians, Vepsas, Estonians) and Saami speaking populations in North eastern Europe (Supplementary Table S3). The highest frequency is detected in Finns (~44%) but it also reaches up to 32% in Vepsas and around 20% in Karelians, Saamis and North Russians. The latter are known to have changed their language or to be an admixed population with reported similar genetic composition to their Finnic speaking neighbors^36^. The frequency of N3a4-B535 rapidly decreases towards south to around 5% in Estonians, being almost absent in Latvians (1%) and not found among Lithuanians. Towards east its frequency is from 1–9% among Eastern European Russians and populations of the Volga-Ural region such as Komis, Mordvins and Chuvashes, but it is absent among Tatars and Bashkirs with an exception of Burzyansky District Bashkirs where the frequency is up to 5% (Supplementary Table S3).

Hg N3a4-B539 (Fig. 3c), on the other hand, is prevalent among Turkic speaking Bashkirs and also found in Tatars but is entirely missing from other populations of the Volga-Ural region such as Uralic speaking Udmurts, Maris, Komis and Mordvins, and in Northeast Europe, where instead N3a4-B535 lineages are frequent. Besides Bashkirs and Tatars in Volga-Ural region, N3a4-B539 is substantially represented in West Siberia among Ugric speaking Mansis and Khantys. Among Hungarians, however, N3a4-B539 has a subtle frequency of 1–4% (Fig. 3d), which is surprising considering their distant location from the core area of N3a4-B539. The sub-clades of N3a4-B539 (B540 and B545) (Fig. 2) have partly overlapping distribution areas with highest frequency peaks of hg N3a4-B540/L1034 (Fig. 3e) in the Ural Mountain region. B540 is more widespread and the most frequent among different sub-populations of Bashkirs in Southern Urals (up to 60%) and in West Siberia among Ugric speaking Mansis and Khantys (up to 27%). It is also present in Uralic speaking Nenets (7%) and Turkic speaking Dolgans (5%) but missing from Uralic speaking Nganasans and Selkups. Although N3a4-B540 is prevalent in Bashkirs it has a considerably lower frequency among neighbouring Tatars (3–5%). B540/L1034 sister-clade B545, however, has a much more localised distribution area – it is present with high frequencies (52%) in Volga-Ural region only among Bashkirs from Sterlibashevsky district of Bashkortostan (Supplementary Table S3). Despite the low sample size, it is notable that both 10^th^ century ancient Hungarian samples from Upper-Tisza region that were derived at Z1936 also belong to the B545 clade^32^. Additionally, contemporary Mansis, who have a high amount of N3a4-B540, lack N3a4-B545. Most intriguingly, similarly to Bashkirs and Tatars both N3a4-B539 sub-clades can be found among Hungarians (Fig. 2, Supplementary Table S3). The frequency of B540 lineage is highest among Sekler Hungarians (around 4%), who today live in Transylvania, Romania. The Sekler are half-million Hungarian speaking people whose historical background is not completely understood. They are distinguished from other Hungarian groups even in the earliest Hungarian chronicles^37^ and form a geographically and genetically isolated sub-population among Hungarians. Csanyi *et al*.^15^ has shown earlier that Sekler Hungarians have hg N3 (1%), but the resolution level used in this study does not allow us to specify further sub-clades. In other Hungarian sub-groups, the frequency of the B540 clade is around 1%. The B545-clade is absent in Sekler Hungarians and is less than 1% in other Hungarian sub-groups (Supplementary Table S3).

To further reveal the inner structure within N3a4 sub-clades we constructed a median-joining network for all Z1936 carrying Y-chromosomes based on 16 STR loci (Supplementary Fig. S2, Supplementary Table S4). The N3a4 network shows a clear clustering of sub-haplogroups. The B535 sub-clade consists of a loose cluster prevalent among Vepsas and Central Russians and a star-like cluster that contains all Finnish, Saami, Northern Russians, Mordovin, as well as the overwhelming majority of Estonian and Karelian samples plus one Ukrainian and Vepsa sample. Such STR pattern indicates a possible SNP-based sub-structure of the clade. The B540/L1034 sub-cluster has a distinguished star-like pattern with a major founder haplotype containing 14 STR haplotypes from different studied populations; Bashkirs, Mansis, Hungarians, Khanty and Tatars. The three Ukrainian, the single Greek and a West Hungarian sample represent a smaller branch. The B545 network did not form a clear sub-cluster in the network, this might be due to the relatively low number of available haplotypes.

Two ancient DNA samples of Hungarian Conquerors derived at Z1936^32^, were included in the network. Their B545 status could not be confirmed, but their STR pattern showed a closer relationship to contemporary Bashkir and Tatar samples than present-day Hungarians.

We tested whether a simple scenario with no gene flow nor admixture can explain the observed frequencies of haplogroup N3a4-B539 in Hungarian Sekler (4%), their neighbouring Indo-European populations (0%) and Southern Ural/West Siberian populations (13%). For that we implemented 3 models: A, B and C (see Material and Methods, Supplementary Fig. S5). Our simplified model shows that Model A depicting the situation with drift alone is almost never accepted (mean 0.04% and standard deviation 0.19%). Although Model B, where drift affects Hungarian Seklers and Indo-Europeans after receiving same external genetic contribution, is more accepted (mean 0.58% and standard deviation 0.73%), it is still significantly rejected (more than 95% of the time). Thus, the other alternative model C, where Southern Ural/West Siberian populations have different contributions in Hungarian and their neighbouring Indo-European populations, is accepted. In all the models we assume that the contribution of N3a4-B539 is coming from Southern Ural/West Siberian populations and the expected frequency of the carriers of the B539 among them is 12.4% (6.8–18% Confidence Interval [CI]; Supplementary Fig. S3). According to Model C the expected contribution of Southern Ural/West Siberian populations to Hungarian populations is 43% (0–89% CI), but the wide CI suggest that we do not have much power in this frequency estimate. Whereas we have much higher confidence in estimating the negligible 3.3% (0–10% CI) contribution of B539 in Indo-European populations.

## Discussion

The comparison of genetic and cultural history of human populations has excited scientists for decades^10,35,38–44^. It is widely accepted among both archaeologists and linguists that the earlier (pre)historical phase of Hungarians points to West Siberia, East of the Ural Mountains^6–9,45–48^ (Fig. 1a), but there is a debate about the potential archaeological cultures involved^3,46^ (Fig. 1b). The first widely accepted station on the early Hungarian migration route is the Kushnarenkovo (6^th^‒8^th^ centuries CE)^49–52^ and the succeeding Karayakupovo cultures (9^th^‒10^th^ centuries CE)^52–54^, with sites mainly found in present-day Bashkortostan, Tatarstan and in the Chelyabinsk area of the Trans-Ural region^8^. Based on the archaeological evidence, a portion of the Hungarians moved to the west in the middle of the 9^th^ century and appeared near the lower reaches of the Dnieper River in present-day Ukraine, where Hungarians are mentioned in historical sources^8^. However, archaeological sites of Chiyalikskaya culture (11^th^–13^th^) support the survival of Hungarians in the Ural region^55,56^. Furthermore, other sources confirmed the survival of Hungarians in the Ural region until the 13th century^45,46^. Also, the eastern Hungarian homeland called *Magna Hungaria* (Great or Ancient Hungary) is referred in the early Hungarian chronicles^48,57^.

Studies based on chrY have shown that the frequency spectrum of different chrY haplogroups and sub-clades varies in large range in different sub-populations^58^, a phenomenon observable also in our study of N3a4 clade (ranging in frequency from 1–59%) (Supplementary Table S3). Compared to earlier study by Feher *et al*.^31^, where shared lineages of hg N3a4-Z1936 between geographically distant but linguistically close Hungarians and Mansi were first reported, present study covers more populations and a wider geographical area (Supplementary Table S3).

While chrY hgs usually show smooth distribution patterns, the particular spatial distribution of hg N3a4-B539 and its sub-clades is important in distinguishing the paternal roots of Hungarians. The eastern roots of hg N have been revealed earlier by comprehensive phylogenetic and phylogeographic study of contemporary hg N lineages by Ilumäe *et al*.^27^. Different hg N lineages among aDNA samples from East Asia and Baikal region show that hg N was frequent and diverse in Neolithic China^59^ and Baikal region^60^ already more than 6000 years ago. N3a4-B539 sub-clades have a well-centred frequency cline in Ural region and West Siberia, but the presence of these clades in only among geographically distant Hungarians and not among any of their neighbours (Fig. 3, Supplementary Table S5) is noteworthy. Simulations enable us to reject a simple random drift model and a single migration model between all Europeans and Southern Ural/West Siberian populations. We also show that there is clearly a direct higher contribution from Southern Urals/West Siberia to Hungarian populations, but the amount cannot be pinpointed using our current data (Supplementary Fig. S3). This might reflect the migration of ancestors of Hungarians from the Ural region to the Carpathian Basin, that is also documented in historical records. The occurrence of hg N3a4-Z1936 among the remains of the individuals from the archaeologically richest 10^th^ century cemeteries of the Hungarian Conquerors in the Carpathian Basin lend support to the Ural region origin of at least part of the Hungarian Conquerors.

It is important to note that there are two different N3a-L708 subgroups that are frequent around the Baltic Sea with a clear south-north frequency gradient tendency^27^. N3a3-VL29 is frequent among Estonians and Latvians and can be detected also among Ukrainians, being the most westward distributed sub-clade among N3a^27^ and therefore finding it in low frequency (0–4%) at the outer borders of distribution zone in different Hungarian sub-populations is expected (Supplementary Table S3). The case of N3a4-B539 and its sub-clades is different: presence of these clades in the Volga-Ural region, in West Siberia and in geographically distant Hungarians is not so easy to explain by gradual frequency cline and without assuming a migration of people (who might have been among the ancestors of the present-day Hungarians).

The split between of B540 and B545 subgroups within Baskhirian, Tatar and Hungarian populations started around 2700–2900 yBP (Fig. 2, Supplementary Table S2) that is in accordance with the recent linguistic data about the divergence of Ob-Ugric and Hungarian languages^61^. It has been proposed that the ‘Ugric Age’ lasted at least until the late Bronze Age in West Siberia and the split between Ob-Ugric and Hungarian from the common proto-Ugric branch of the Uralic language tree occurred during the first centuries of the first millennium BC^9^, but the recent linguistic reconstructions of the Uralic language tree give much broader borders for the divergence of Ugric clade (4900‒1700 yBP)^61^. The time-frame is the same for the cooling climate in West Siberia with its peak at the 9^th^ and 8^th^ CE which could have resulted in the movements of several West Siberian populations^61,62^.

Although the frequency of hg N3a4-B539 is subtle among present-day Hungarians, it is possible that ancient Magyars who lived in the Ural Mountain region had a significantly higher proportion of chrY hg N, since the Z1936 lineage was found from 5 individuals out of 19 (26,3%) in the archaeologically richest Hungarian late 9^th^-early 10^th^ century cemeteries^32^. This frequency is quite similar to Z1936 > B539 frequencies found among various Khanty, Mansi and Bashkir groups (Supplementary Table S3).

The homeland of ancient Hungarians around the Ural Mountain region, and the Hungarian affinities of Kushnarenkovo and Karayakupovo cultures is widely accepted among researchers^47,49–54^. Further studies of chrY and autosomal diversity in ancient samples of the representatives of those cultures could also provide new insight into the demographic history of the Hungarians.

## Material and Methods

### Whole Y-chromosome sequencing and phylogeny reconstruction

For reconstructing the phylogeny of N3a4 clade we included 8 whole chrY sequences published earlier in Karmin *et al*.^63^, 6 sequences published in Ilumäe *et al*.^27^, 2 sequences published in Wong *et al*.^64^, and 17 new sequences from this study. Two N3a2 and six N3a4 sequences were generated with Complete Genomics (Mountain View) technology at 40x coverage. Six published and 17 samples from current study were sequenced at Gene By Gene by using the commercially available “BigY” service. The complete chrY sequences have been deposited to European Nucleotide Archive with the accession numbers ERS2768175 to ERS2768191 (study accession PRJEB28776).

Mapping of fastq files was done using BWA-MEM (v0.7.12)^65^ and the human reference hs37d5. Read duplicates were removed using Picard (v2.0.1)^66^ followed by realignment around known indels and base quality score recalibration (BQSR) using GATK (v3.5)^67^. Variant calling was performed with GATK tool HaplotypeCaller. Filtering of the raw VCF files produced by GATK was done using bcftools (v1.4)^68^. We merged both the Illumina and Complete Genomics filtered data sets using CombineVariants from GATK (v3.8)^67^. We extracted the effective overlap between the two data sets by masking out all the positions with 5% or higher proportion of missing genotypes in either the Illumina or the Complete Genomics data sets. We also masked out regions with poor mappability as described in Karmin *et al*.^63^, resulting in a final total of 9.7 Mb of sequence analyzed.

We implemented the software package BEAST v.1.7.5^69^ to reconstruct the hg N3a4 phylogenety and to estimate coalescent times by using two N3a2 samples as an outgroup. We used a Bayesian skyline coalescent tree prior, the general time reversible (GTR) substitution model with gamma-distributed rates, and a relaxed lognormal clock. The results were visualized in Tracer v.1.4. As a calibration point for coalescent time estimation we used an age for hg N3a2′6 of 7113 years (95% CI = 6,076–8,252)^27^. The N3a4 phylogenetic tree and the mutation list (Supplementary Table S1) were manually annotated (Supplementary Fig. S4).

Throughout the study nomenclature of Karmin *et al*.^63^ and its updates in Ilumäe *et al*.^27^ was followed.

The list of sample ID labels used in this study is provided in Supplementary Table S6. All samples were obtained from unrelated volunteers who provided informed consent in accordance with the guidelines of the relevant collaborating institutions and approved by the Research Ethics Committee of the University of Tartu (approval 228/M-40).

### Sampling and genotyping

This study includes earlier published datasets from different sources, altogether about 5000 samples from 46 populations^27,31,70–73^ including 4 different Hungarian sample-sets of different sub-populations (Supplementary Fig. S1, Supplementary Table S3). 329 samples which belonged to N3a4 clade were updated to a higher level of phylogenetic resolution within the inner-structure of N3a4. Two samples were previously assigned using chrY short tandem repeats (Y-STRs)^72^. For genotyping branch defining SNPs from N3a4 sub-clades we designed primers with Primer3 software^74,75^. Primer specificity was first checked with Primer-BLAST^76^ and GenomeTester v.1.3 software^77^ and verified by Sanger sequencing. All the samples were hierarchically genotyped using Sanger sequencing. The specifications for the used markers can be found in Supplementary Table S7.

The frequency distribution maps of hg N3a4 and its sub-clades were created with Surfer® (v.8, Golden Software, Inc, Golden, CO, USA). Data used for generating the maps is presented in Supplementary Tables S3 and S4.

We used data from 16 chrY STRs genotyped in 128 samples in this study and merged with previously published datasets to construct the phylogenetic network (Supplementary Fig. S2, Supplementary Table S4). The network was constructed with Network 4.6.1.1 software (Fluxus-Engineering) by applying median joining algorithm.

### Model selection based on resampling procedure

To test if a scenario with no gene flow or admixture is sufficient to explain the observed frequency of haplogroup N3a4-B539 in Hungarian Sekler, neighbouring Indo-European and Southern Ural/West Siberian populations we implemented a resampling approach, similarly to the one implemented in Barbieri *et al*.^78^ and Marks *et al*.^79^. In detail, we assumed constant population size and no mutation (as we wanted to see how the haplogroup frequency drifted within population and having mutation would not change the frequency of the particular haplogroup). All the procedure was performed using python, numpy version 1.14.3 and pandas 0.20.3^80,81^. We set the effective population size of all populations 5000 individuals and two “alleles”: one haplogroup N34-B539 denoted as 1 and all other haplogroups denoted as 0. In every generation, we randomly resampled (with replacement) N (N = effective population size) number of haplotypes from the previous generation (using numpy.random.choice). In order to simulate single migration event or admixture, a randomly selected α proportion of alleles from one population was moved into another. At final generation, we randomly selected 2677, 95, 957 samples, reflecting the sample size of Indo-European, Hungarian Sekler and Southern Ural/West Siberian, respectively.

Finally, we have evaluated the frequency of haplotype “1” (N34-B539) and retained the simulation as successful (which will be later used for the success rate of a given model) only if the simulated “1” frequency was within 2SD of the observed one, estimated as follows:

$$tolerance=2SE=2\times \sqrt{\frac{p(1-p)}{2n}}$$

where: SE =  standard error, p = haplogroup frequency, n =  sample size

When the observed frequency was  = 0, we set it to 1*/n* + *1*.

We implemented the following three models A, B and C (Supplementary Fig. S5).

In Model A, we want to check if the haplogroup N3a4-B539 frequency in Indo-Europeans (0%), Hungarian Sekler (4%) and Southern Urals/West Siberians (13%) can be the result of genetic drift alone. In doing so, we simulated three populations which diverged 100 generations ago (3,000 years assuming a generation time of 30 years) with a starting frequency for N3a4-B539 randomly generated from a uniform distribution with min = 0 and max = 1. As in model A, our null hypothesis is that the haplogroup frequencies of N3a4-B539 can be explained by random drift alone, we retained only those simulations which have final haplogroup frequency within two standard errors of the one observed in Siberia (assuming by fixing one haplogroup frequency we can explain the others by drift alone). We then calculated the success rate as mentioned above to know if the null hypothesis can be rejected.

In Model B, we want to check if the lack of N3a4 haplogroup observed in Indo-European, but not Hungarian populations might be the result of genetic drift after having the same amount of genetic contribution from an external source (e.g Southern Ural/West Siberians). We simulated Southern Ural/West Siberian and Indo-European populations separately. Southern Ural/West Siberian population already has haplogroup N3a4 (with unknown proportion as above) whereas both Indo-European and Hungarian Sekler populations do not. We simulated one single admixture event from Southern Ural/West Siberian to both Indo-European and Hungarian Sekler populations occurring 30 generations ago, with same unknown amount of admixture [using numpy.random.random_sample] and then randomly drifted for 30 generations. Here our null hypothesis was a single admixture event from Siberia in European populations (both Indo European and Hungarian) can explain the haplogroup frequencies. Thus, we chose only those events which have the final frequency of 13% in Southern Ural/West Siberia with Hungarian frequency of 4% (within tolerance limit). We then calculate the success rate of model B.

To compare between models, we chose such simulations 100 times for every model. We then calculated at how many times the populations have similar haplogroup frequency as modern observed populations. We repeated this process 100 times to get mean and standard deviation (which is essentially 10000 simulations).

To provide possible parameters explaining the dynamics resulting the observed N3a4 haplogroup frequencies, we simulated an additional model.

In Model C, we wanted to calculate the parameters and the confidence interval to see how much power we have for our accepted model. This model is similar to Model B but instead of same admixture proportion from Southern Ural/West Siberia to Indo-Europeans and Hungarians, we put two independent amounts. We only chose those simulations where final frequency is within two SDs from the observed (thus the success rate is 100 percentage). We repeated the procedure 1000 times to calculate the mean and confidence interval for those unknown parameters.

## Supplementary information

Supplementary information

Supplementary Table S1

Supplementary Table S2

Supplementary Table S3

Supplementary Table S4

Supplementary Table S5

Supplementary Table S6

Supplementary Table S7
