# Supplementary material for: Y-chromosomal connection between Hungarians and geographically distant populations of the Ural Mountain region and West Siberia
Source: Sci Rep. 2019 May 24;9:7786. doi: 10.1038/s41598-019-44272-6 (PMC6534673; doi:10.1038/s41598-019-44272-6)
Supplement: Supplementary file 1 — Supplementary information [file 41598_2019_44272_MOESM1_ESM.pdf]

Y-chromosomal connection between Hungarians and geographically distant populations of the Ural Mountain region and West Siberia

Helen Post[1,8]* and Endre Németh[2]*, László Klima[3], Rodrigo Flores[1], Tibor Fehér[2], Attila Türk[4], Gábor Székely[2], Hovhannes Sahakyan[1,5], Mayukh Mondal[1], Francesco Montinaro[1], Monika Karmin[1], Lauri Saag[1], Bayazit Yunusbayev[1,6], Elza K. Khusnutdinova[6,7], Ene Metspalu[1], Richard Villems[1,8], Kristiina Tambets[1], Siiri Rootsi[1]

[1]Institute of Genomics, Estonian Biocentre, University of Tartu, Tartu 51010, Estonia

[2]Hungarian Natural History Museum, Department of Anthropology, Budapest 1083, Hungary

[3]Eötvös Loránd University Budapest, Department of Finno-Ugric Studies, Budapest 1088, Hungary

[4]Pázmány Péter Catholic University, Faculty of Humanities and Social Sciences, Department of Early Hungarian and Migration Period Archaeology, Piliscsaba 2087, Hungary

[5]Laboratory of Ethnogenomics, Institute of Molecular Biology of National Academy of Sciences, Yerevan 0014, Armenia

[6]Institute of Biochemistry and Genetics, Ufa Scientific Center of RAS, Ufa, 450054 Russia

[7]Department of Genetics and Fundamental Medicine, Bashkir State University, Ufa, 450054 Russia

[8]Department of Evolutionary Biology, Institute of Molecular and Cellular Biology, University of Tartu, Tartu 51010, Estonia

* These authors contributed equally to this work

Corresponding Author: Helen Post

Institute of Genomics
23 Riia Street, 51010, Tartu, Estonia
helen2po@ut.ee

Figures

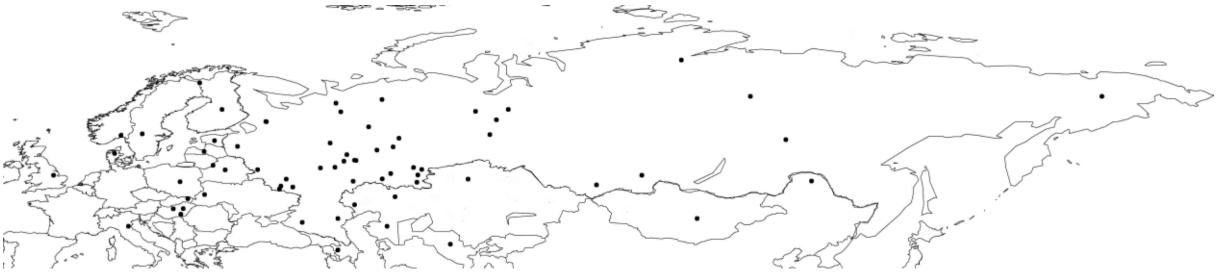

Figure S1. Map with sampling points.

Background maps from Surfer® (v.8, Golden Software, Inc, Golden, CO, USA).

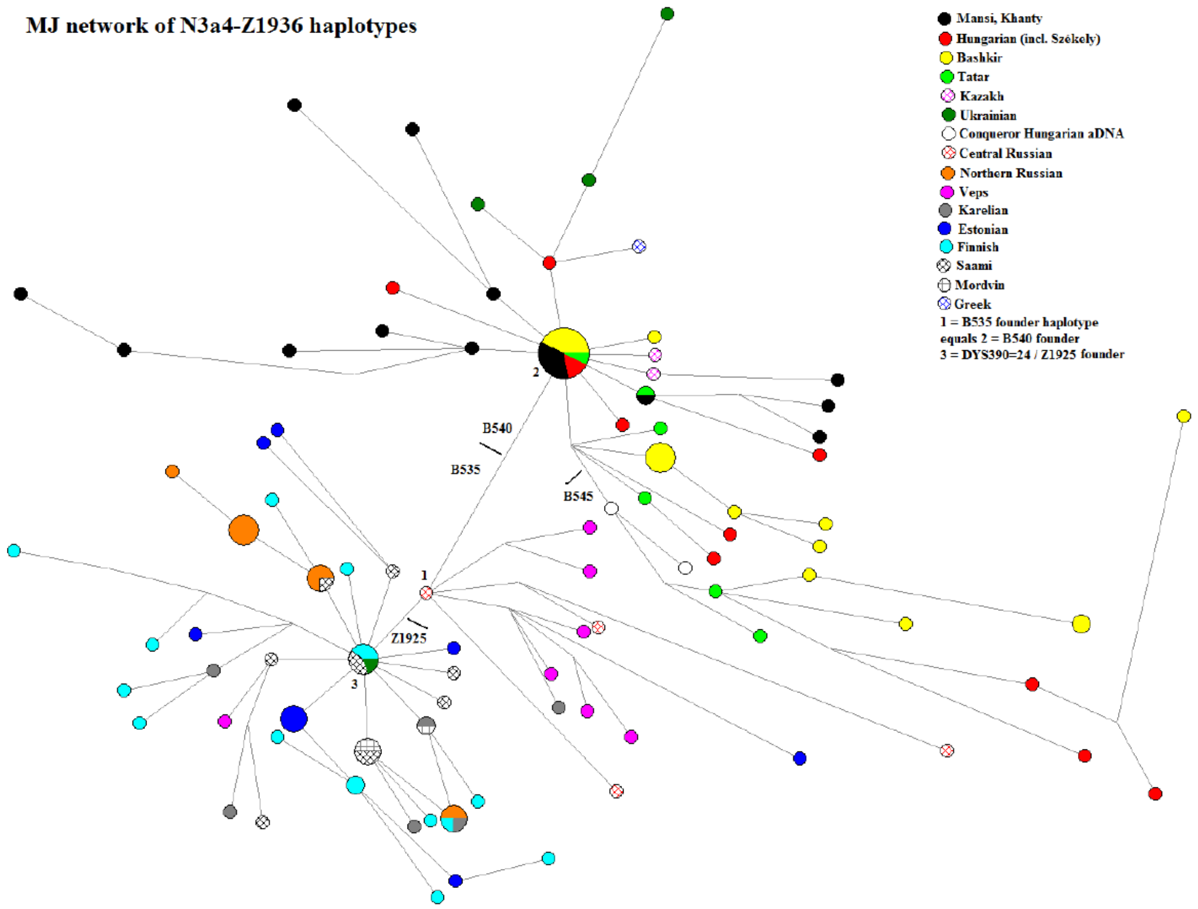

Figure S2. Network of N3a4 clade.

The network was constructed with Network 4.6.1.1 software by applying median joining algorithm and using haplotypes of 16 Y STRs (Supplementary Table S4).

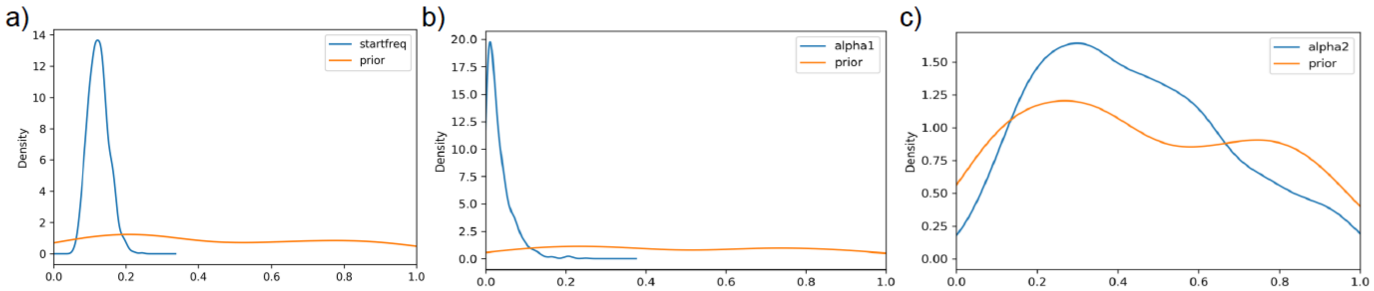

Figure S3. The posterior and prior distributions from Model C.

a) Haplotype frequency of Siberian populations which contributed to Europeans and Hungarians. b) Admixture proportion from Siberian to Europeans and c) Admixture proportion from Siberian to Hungarians. Posterior distribution in blue and prior distribution in orange.

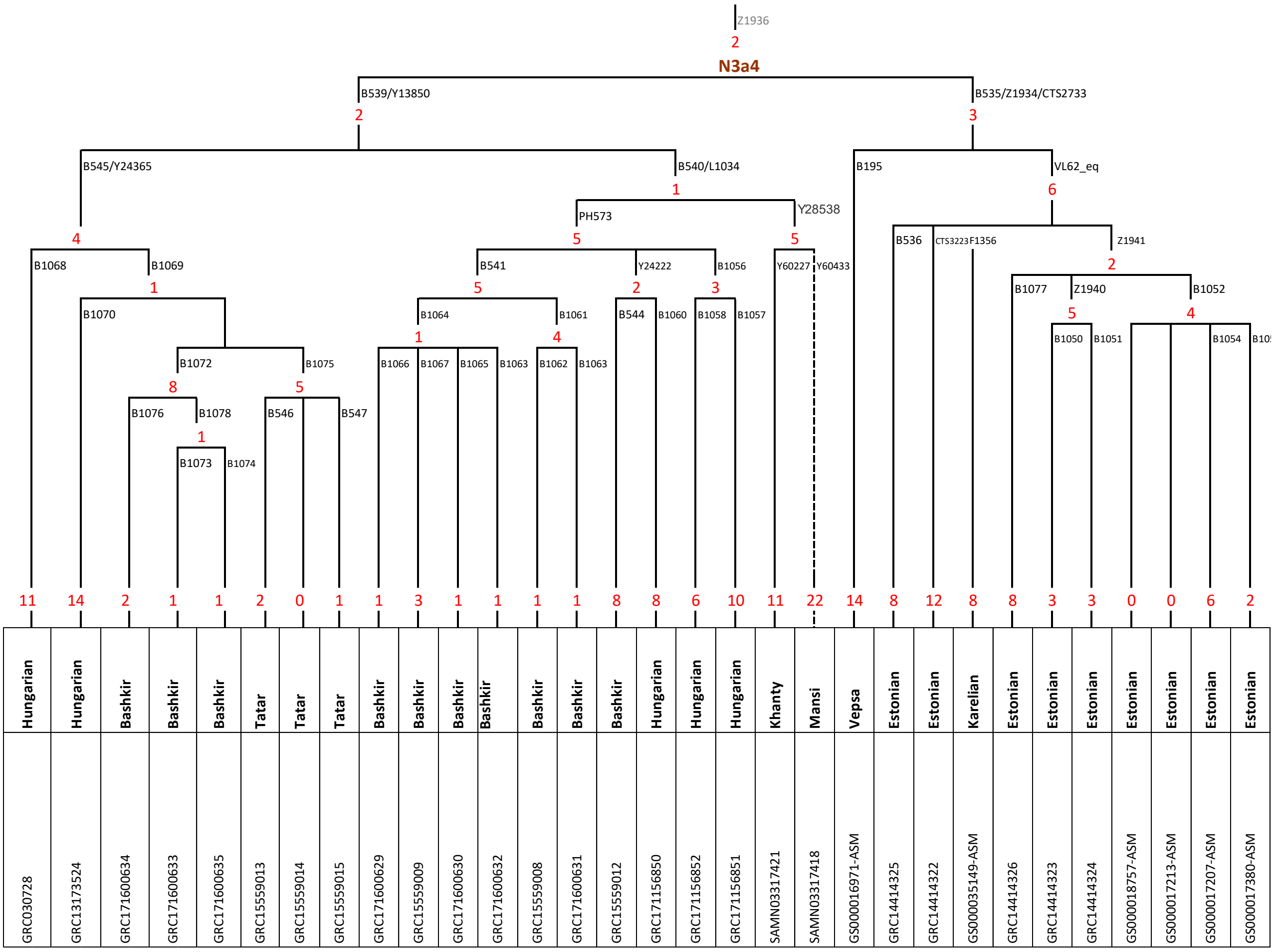

Figure S4. Manually annotated N3a4 phylogenetic tree.

The N3a4 phylogenetic tree and the mutation list were manually annotated. Refined topology of Y-chromosome haplogroup N3a4 based on re-sequencing in 33 individuals described in Supplementary Table S7. Mutation counts (in red) and branching defining markers (included in counts and shown in grey colour) are shown on the branches.

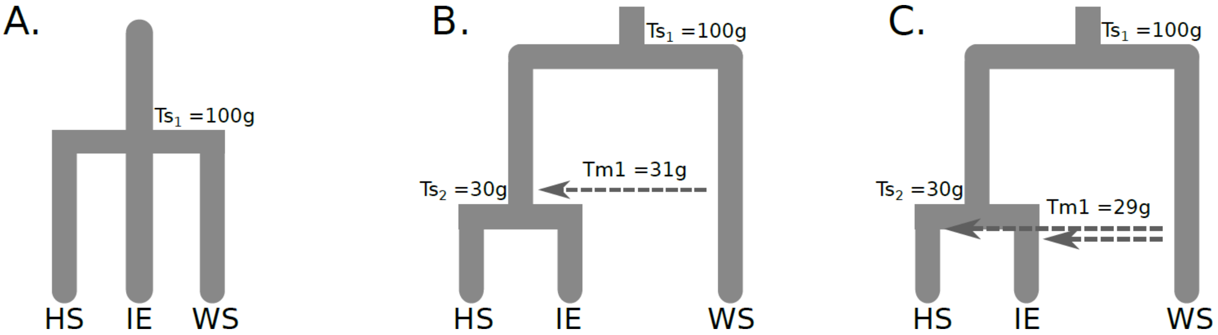

Figure S5. Overview of the simulated models A, B and C using the resampling approach. Abbreviations: Ts: Time of split, Tm: Time of migration, HS: Hungarian Sekler, IE: Indo-European, WS: West Siberian.

Tables

Table S1. Mutation list for phylogenetic tree of N3a4

Table S2. Age estimates of N3a4 sub-clades according to Figure 2

Table S3. Geographic distribution of hg N2a1 and N3 subclades

Table S4. N3a4 STR haplotypes

Table S5. Locations of populations for local B539 surfer map

Table S6. List of re-sequenced samples and their geographic affiliations

Table S7. Specifications for SNPs used to genotype population samples
